# Treatment strategy for patient with non-syndromic tooth agenesis: a case report and literature review

**DOI:** 10.1186/s12903-024-04613-y

**Published:** 2024-07-25

**Authors:** Tianfeng Ouyang, Dong Chen, Zeli Ma, Xin Li, Ge Cao, Lin Lin, Ming Zeng, Ting Chen

**Affiliations:** Department of Stomatology, Nanfang Hospital, Southern Medical University, 1838 N Guangzhou RD, Guangzhou, 510080 Guangdong People’s Republic of China

**Keywords:** Non-syndromic tooth agenesis, Ectodysplasin A, Case report

## Abstract

**Background:**

Non-syndromic tooth agenesis (NSTA) is a type of ectodermal dysplasia (ED) in which patients with non-syndromic oligodontia may only affect teeth. No pathological findings were found in other tissues of the ectodermal. Herein, we report a case of a NSTA patient with severe dental anxiety and poor oral health.

**Case presentation:**

A 5-year-old boy without systemic diseases presented as a patient with oligodontia, extensive caries, and periapical periodontitis. Molecular genetic analysis found a mutation in the Ectodysplasin A (EDA) gene, confirming the diagnosis of NSTA.

**Conclusion:**

Tooth agenesis (TA) is the most common ectodermal developmental abnormality in humans. Non-syndromic oligodontia patients often seek treatment in the department of stomatology. Because of their complex oral conditions, these patients should be provided with a systematic and personalized treatment plan.

## Background

Tooth agenesis (TA) is a common disease of dental development anomalies in dental clinics, categorized by the number of missing teeth into hypodontia, oligodontia, and anodontia. There are various causes of congenitally absent teeth, including genetic factors, trauma, infection, certain drugs taken during pregnancy, or defects of uncertain etiology [1]. Most cases are genetically determined [2], with mutations in genes like EDA, WNT10A, PAX9, AXIN2, and MSX1 responsible for the condition [3]. Located on chromosome Xq12-13.1, the EDA gene encodes a 391 residue protein [4]. The EDA gene mutation can lead to X-linked hypohidrotic ectodermal dysplasia (XLHED) and non-syndromic tooth agenesis (NSTA). NSTA patients exhibit dental abnormalities, while other ectodermal organs, such as hair, nails, and glands, do not show pathological changes [5].

Here, we describe a 5-year-old boy with non-syndromic tooth agenesis (NSTA, OMIM#313,500) who carried a missense mutation (c.1013 C > T, Thr338Met) in the EDA gene by whole exome sequencing (WES). His oral issues were addressed through a general anesthesia procedure. We improved his occlusion and masticatory function with removable partial dentures.

## Case presentation

**Fig. 1:**
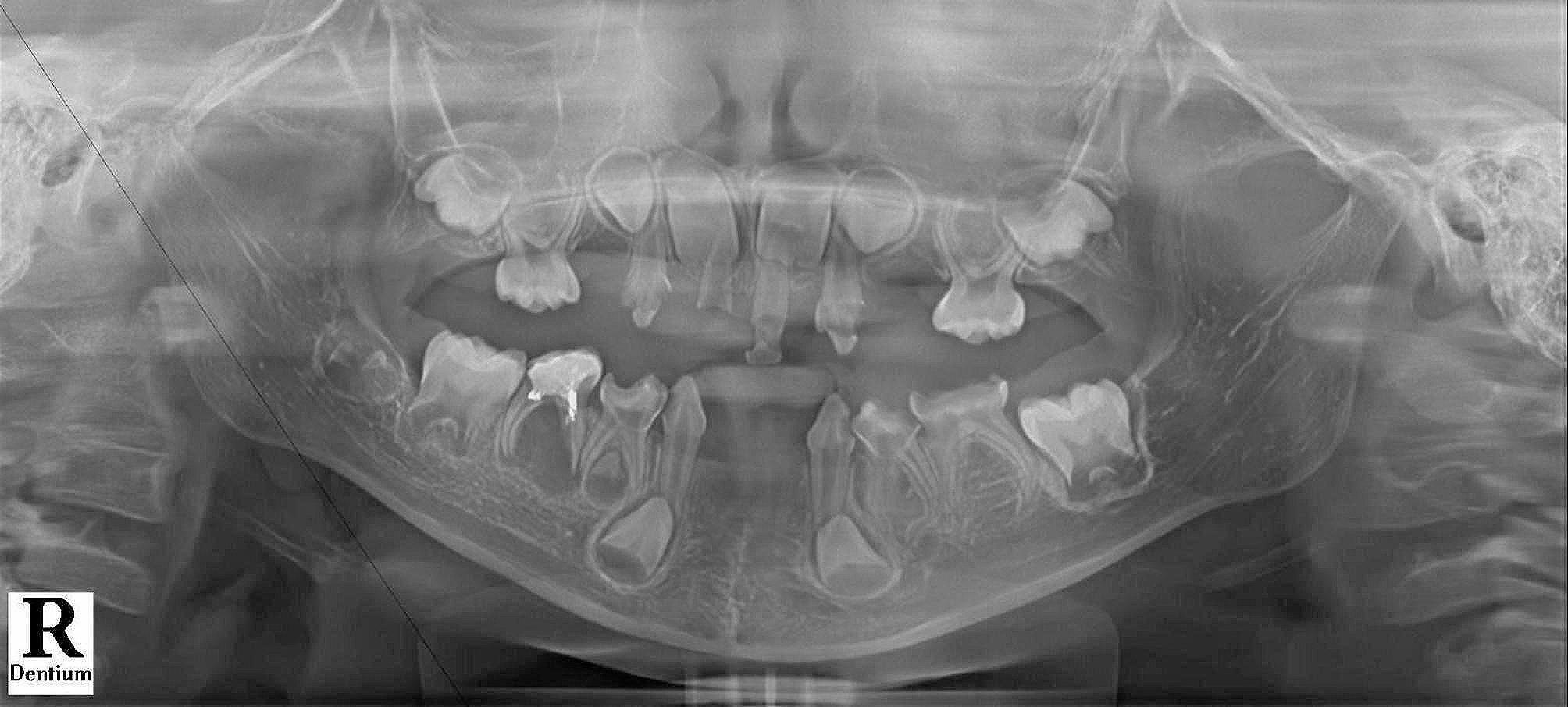
Initial panoramic radiograph of the patient

A 5-year-old boy with cavities and toothache visited the Department of Pediatric Dentistry. The patient comes from a non-consanguineous family. He was 105 centimeters tall and weighed 15.5 kg. Upon examination, it was found that he was in mixed dentition stage; seven deciduous teeth were missing, and the other deciduous teeth were carious. Tooth #51 was a residual root. Tooth #85 had pain upon percussion. Previously, this tooth was filled with glass ionomer cement (GIC) for the crown in another hospital. Panoramic radiographs confirmed the loss of more than 16 permanent teeth germs. Mandibular deciduous and permanent incisors are absent, while only the germ of tooth #44 existed. The shapes and sizes of the residual teeth were regular. A broad low-density shadow was observed in the apical region and the furcation of tooth #85 (Fig. 1).

During the initial visit, the patient was categorized as Frankl I. He offered strong resistance and was anxious about dental treatment. With the use of protective stabilization, the patient’s body movements were restricted. As a result, tooth #51 was extracted under local infiltration anesthesia in the clinic. Despite attempts at behavioral management, such as tell-show-do, positive reinforcement, and distraction, the patient continued to be uncooperative during subsequent treatments. Given that the patient had multiple teeth requiring treatment and offered stronger resistance to oral therapy, we, with the consent of his parents, addressed his oral problems under general anesthesia.

**Fig. 2:**
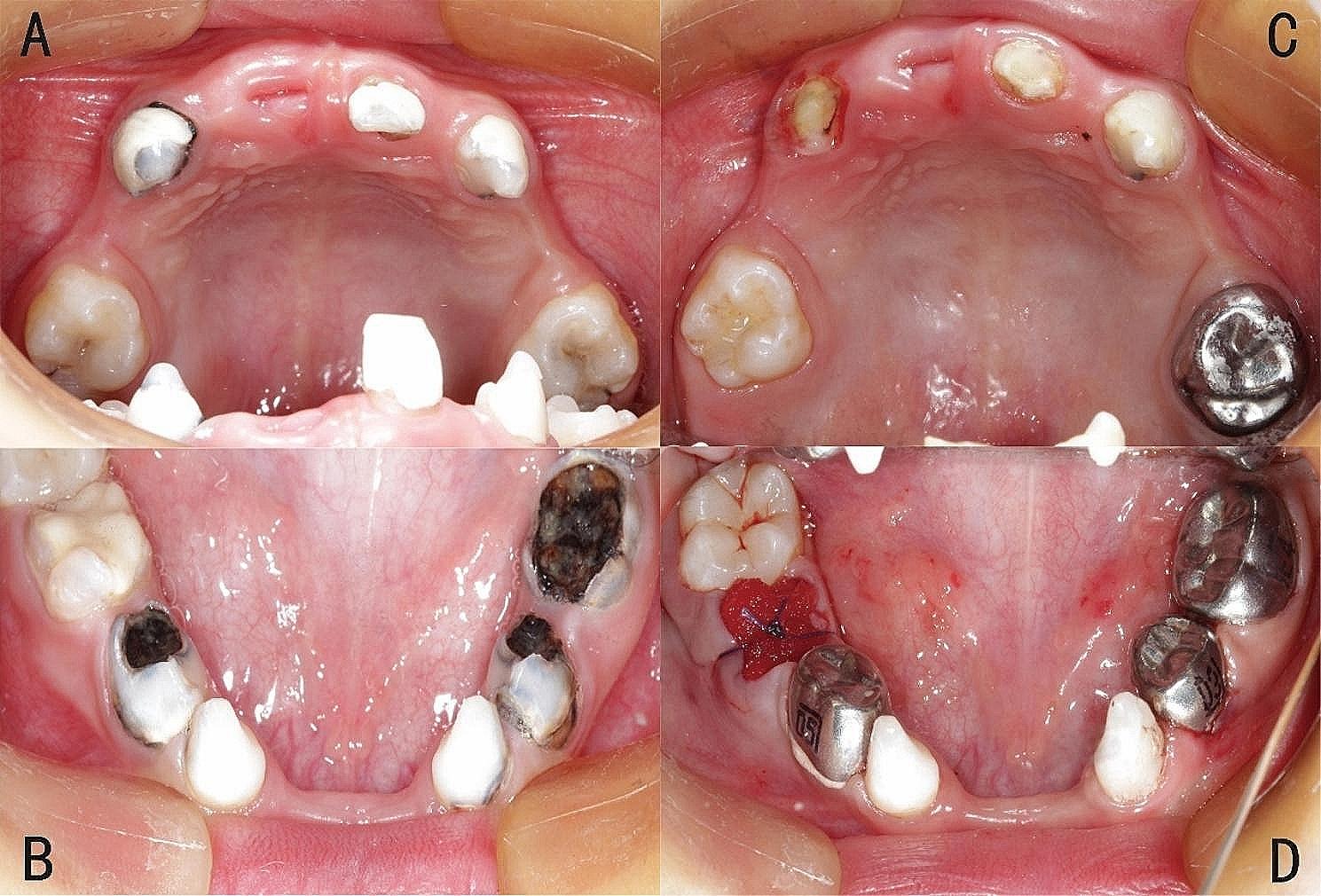
Preoperative intraoral photographs of the patient. **A**: Upper occlusal view of pre-operation. **B**: Lower occlusal view of pre-operation. **C**: Upper occlusal view of post-operation. **D**: Lower occlusal view of post-operation

The operation was successful, and a total of 10 affected teeth were treated under dental general anesthesia. Teeth #53, #61, #63, and #73 underwent root canal therapy (RCT) and were filled with GIC. Tooth #55 was restored with composite resin. Tooth #65 had a large defect in the distal surface after caries removal, so we restored it with a stainless steel crown (SSC). Additionally, teeth #74, #75, and #84 were restored with SSC after RCT. Finally, tooth #85 was extracted due to severe inflammation and a poor prognosis after RCT (Fig. 2).

**Fig. 3:**
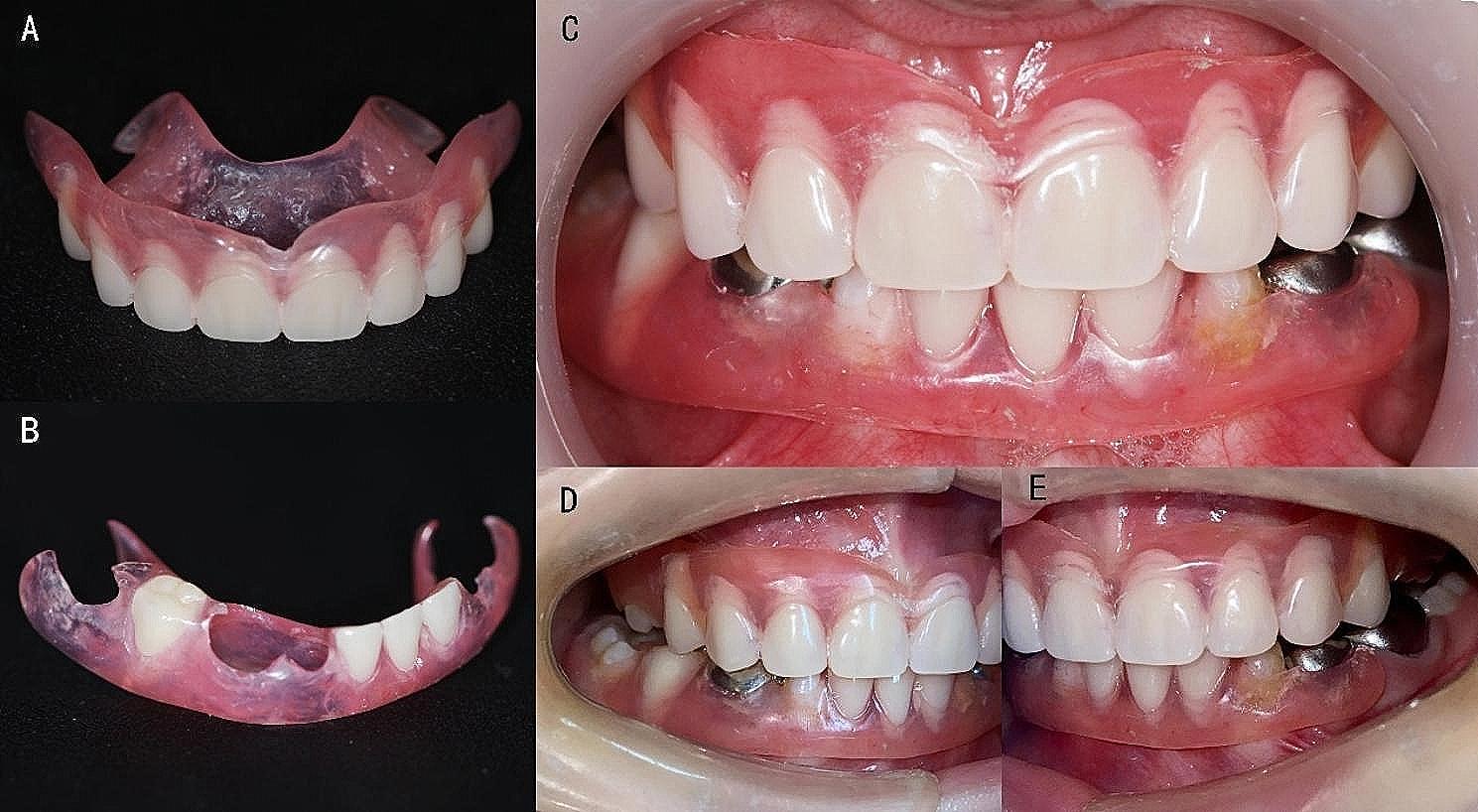
View of the removable partial dentures and intraoral photograph of the removable partial dentures. **A**: Upper jaw of the removable partial denture. **B**: Lower jaw of the removable partial denture. **C**: Frontal facial view. **D**: Right occlusal view. E: Left occlusal view

**Fig. 4:**
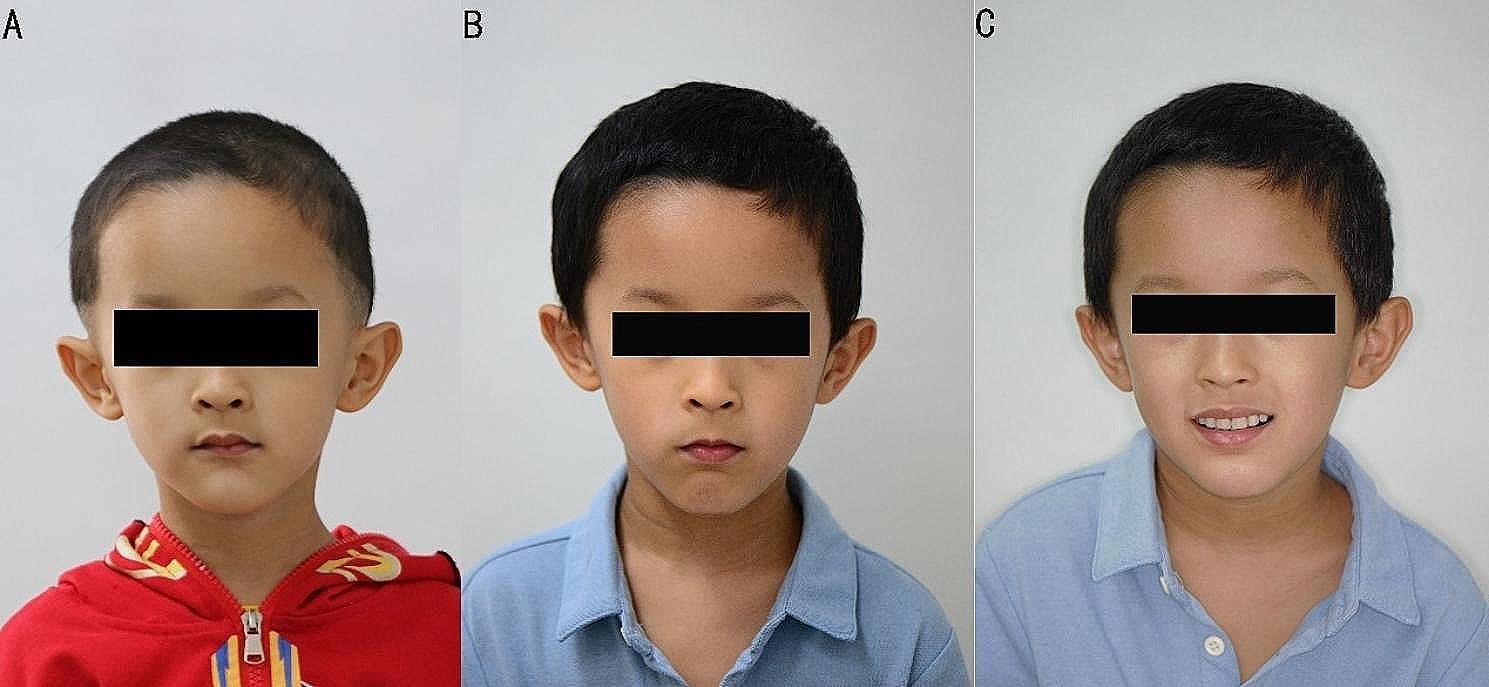
Initial and post-operative facial view of the child. A: Frontal facial view of initial visit. B: Frontal facial view of post-operative. C: Smile frontal facial view of post-operative

The patient came back to the clinic three months later with positive feedback. The teeth were in good condition after treatment. The Department of Prosthodontics recommended a transitional removable partial denture to restore the patient’s oral functions. The dentures were well-seated (Fig. 3). The boy and his parents were informed that the dentures might be uncomfortable in the first few months and should be replaced according to the development of his jaws. Five more months later, he was 112 centimeters tall and weighed 21.2 kg. The condition of the dentures seemed good. His facial fullness was significantly improved (Fig. 4). However, there was no obvious development of the jaws. Additionally, the patient had poor oral hygiene and some supragingival calculus around the abutments. Oral hygiene instructions were provided.

**Fig. 5:**
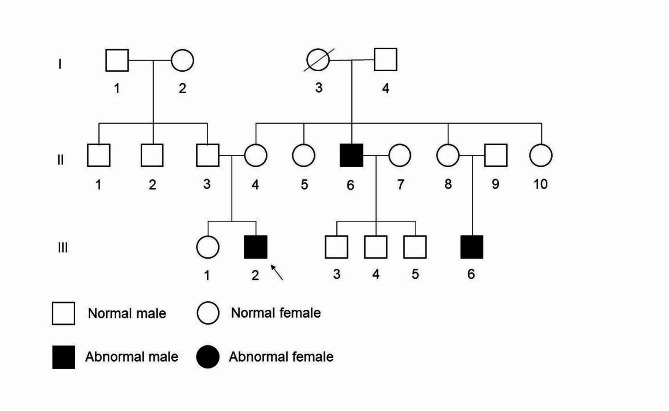
Pedigree of this family

Upon delving into the family history of the proband (III2), we learned that his uncle (II-6) and cousin (III-6) also had similar clinical features. According to the genogram (Fig. 5), the genetic pattern of his family was the recessive inheritance of the X chromosome. To delve deeper into the role of genetic factors in congenitally absent teeth, we obtained blood samples from the proband (III2) and his parents (II3 and II4) and sequenced genes mutation by WES. The blood of other relatives in this family was not collected because of their unwillingness.

The mutation of SNP and INDEL was meticulously detected and filtered by Sentieon DNA seq software. It identified the mutant gene as EDA. The results showed a non-synonymous C to T transition at the nucleotide 1013 in exon 8 of EDA (C. 1013T). It caused the change of the amino acid at position 338 from a hydrophilic threonine (Thr) to a hydrophobic methionine (Met) (P.T338M). This mutation site was reported as early as 2008 [6].

## Discussion

Ectodysplasin-A (EDA) is a 391 residue protein encoded by the EDA gene [7]. Located on the X chromosome, the EDA gene is prone to mutation, which can lead to XLHED, while partial site mutation can also lead to NSTA [5]. During tooth development, EDA is continuously expressed in the dental epithelium from the epithelial thickening to the bud stage It continues to be expressed until the end of the cap stage [7]. EDA is a type II transmembrane protein with four functional domains: an N-terminal intracellular domain, a furin protease recognition sequence, a collagen-like repeat domain, and a C-terminal TNF homology domain [5]. Any change in one of these domains can lead to errors in the protein encoded by the EDA gene, with mutations in the TNF homology domain occurring most frequently. This case report describes a NSTA caused by EDA non-synonymous mutation, As early as 2008 [6], the site of mutation was reported to be in the TNF domain of the EDA protein. Results in the amino acid at position 338 change from hydrophilic Thr to hydrophobic Met, which threatens the stability of the EDA protein and affects corresponding functions [8]. Pan et al. [9] studied the role of EDA in human dental pulp stem cells (hDPSCs) and found that the odontogenic differentiation ability of hDPSCs transfected with mutant EDA lentivirus was reduced, which revealed the possible mechanism of abnormality of tooth number in patients with EDA mutation.

In his family, three members exhibited congenital TA (male 3), affecting mainly the mandibular central and lateral incisors as well as the maxillary lateral incisors, without missing first molars. This tooth phenotype was consistent with the tooth phenotypes of the previously reported cases at this mutation site [6, 8, 9]. Zhang et al. [10] found similar results after calculating the tooth loss sites of 84 patients with the EDA gene mutations. Interestingly, the tooth-missing phenotypes of the EDA gene mutations are roughly symmetrically distributed. Similar to the EDA gene mutations, other gene mutations involved in tooth development also result in the loss of specific teeth sites. Zhou et al. [11] found that the PAX9 gene mutation significantly increased the incidence of molar missing, which led to a lower percentage of agenesis in mandibular premolars. MSX1 mutations tend to be absent in premolars, and the mutation of WNT10A and AXIN2 probably affected maxillary and mandibular second premolars [11]. Therefore, patients with similar dental phenotypes may be considered for mutations in related genes. WES can be used to explore the genetic causes of their disease [12], offering a potential breakthrough in our understanding of TA.

The patient, like other NSTA patients, had an absence of most deciduous and permanent teeth, but did not show any abnormal manifestations of other ectodermal organs such as hair, nails, and glands. Moreover, there was no change in the tooth phenotype, which can occur in patients with syndromic congenital. Lexner et al. [13] observed that the anterior teeth of hypohidrotic ectodermal dysplasia (HED) patients were mostly conical or tapered, and the majority of the posterior tooth roots were fused or conical. These phenomena were also observed in other case reports on HED [14–16]. In this case, despite the decreased number of teeth, the appearance of the crown did not change. Sonnesen et al. [17]also found that the upper cervical spine morphology and craniofacial morphology of XLHED patients were different from that of non-syndromatic ectodermal dysplasia. The child did not undergo relevant examination due to the request of his parents. Therefore, his morphologies were not compared with those of XLHED patients.

Dental anxiety (DA) is a general state of apprehension regarding possible negative events or outcomes related to dental treatment [18]. Children with DA often exhibit strong resistance and difficulty in behavior management. The patient had negative dental experiences in another hospital and therefore exhibited DA. Despite repeated attempts at behavior management, the patient was still unable to cooperate with the treatment. The patient was assessed as Frankl I, and his resistance to dental treatment had increased. As the effectiveness of oral sedatives was uncertain, we recommended solving the dental problems under general anesthesia. A study has indicated that oral therapy under general anesthesia can reduce anxiety in children aged 3–5 years [19]. Despite higher costs, the long-term curative effect after treatment is reliable [20]. Furthermore, the long-term dental treatment effects of children under general anesthesia were found to be superior to those treated with protective stabilization [21]. Zhao et al. [22] after general anesthesia to solve dental problems, the BMI of children will improve significantly. It was also observed in this case.

Due to his patient having congenital oligodontia and other dental problems, the patient had lower height and weight than children of the same age group as well as poor mandible development. Primarily, we intended to restore his masticatory function after general anesthesia. AlNuaimi et al. [23] used a fixed bridge to repair the missing mandibular teeth area of a child with HED. They believed that the fixed bridge could slow down the absorption of the alveolar ridge and better improve the masticatory function. Montanari et al. [24] selected implant-supported overdentures to restore the HED patient’s appearance and oral function, and the positive effect was observed in the long-term follow-up. Considering that the patient was in the growth stage with first molars and canines that did not fully erupt, it was appropriate to use the removable partial denture to restore function, maintain the space, and promote the growth and development of the jaws [15].

The use of removable dentures not only facilitates the development of normal dietary habits but also enhances the patient’s social integration [25]. For children with oligodontia and ED, the adapted removable denture can offer a comfortable mobility experience while maintaining the periodontal condition of the abutment teeth during primary and mixed dentition stage [26, 27]. Therefore, it is crucial, as suggested by Pigno et al. [28] to consider prosthetic rehabilitation as early as possible, with a recommended recall schedule of 6–12 months until jaws growth is complete. Further extended follow-up is required to observe the long-term effects of wearing a removable partial denture on the thin alveolar ridge of the missing teeth areas.

## Conclusions

Patients with ectodermal dysplasia, often accompanied by tooth agenesis, present a complex oral situation. However, we can seek solutions to restore their oral functions after a comprehensive analysis of their dental situation and multidisciplinary consultation. In this case, the patient suffered from non-syndromic tooth agenesis. The functional studies on the mutation site have been reported. Despite his severe dental anxiety and difficulties in behavioral management, his dental problems were successfully addressed under general anesthesia. His dentures have proven effective, and the development of his jaws requires long-term follow-up. It is expected that he will experience improved physical development, mental health, and quality of daily life in the future.

## Data Availability

The datasets generated during the current study are available from the corresponding author on reasonable request.

## Abbreviations

NSTA: Non-Syndromic Tooth Agenesis
ED: Ectodermal Dysplasia
EDA: Ectodysplasin A
WES: Whole Exome Sequencing
TA: Tooth Agenesis
XLHED: X-Linked Hypohidrotic Ectodermal Dysplasia
GIC: Glass Ionomer Cement
RCT: Root Canal Therapy
SSC: Stainless Steel Crown
Thr: Threonine
Met: Methionine
hDPSCs: Human Dental Pulp Stem Cells
HED: Hypohidrotic Ectodermal Dysplasia
